# Galectin‐3 exacerbates ox‐LDL‐mediated endothelial injury by inducing inflammation via integrin β1‐RhoA‐JNK signaling activation

**DOI:** 10.1002/jcp.27910

**Published:** 2018-12-10

**Authors:** Xiumei Chen, Jianzhong Lin, Tingting Hu, Zhiyun Ren, Linnan Li, Irbaz Hameed, Xiaoyu Zhang, Chen Men, Yan Guo, Di Xu, Yiyang Zhan

**Affiliations:** ^1^ Department of Geriatric Cardiology The First Affiliated Hospital of Nanjing Medical University Nanjing China; ^2^ Department of Urology and Central Laboratory BenQ Medical Center, The Affiliated BenQ Hospital of Nanjing Medical University Nanjing China; ^3^ Department of Cancer Research The First Clinical Medical College, Nanjing Medical University Nanjing China; ^4^ Department of Cancer Research Academy of Pediatrics, Nanjing Medical University Nanjing China; ^5^ Department of Cardiothoracic Surgery New York Presbyterian Hospital Weill cornell Medicine New York New York

**Keywords:** atherosclerosis, endothelial injury, Galectin‐3, inflammation, integrin β1, JNK

## Abstract

Oxidized low‐density lipoprotein (Ox‐LDL)‐induced endothelial cell injury plays a crucial role in the pathogenesis of atherosclerosis (AS). Plasma galectin‐3 (Gal‐3) is elevated inside and drives diverse systemic inflammatory disorders, including cardiovascular diseases. However, the exact role of Gal‐3 in ox‐LDL‐mediated endothelial injury remains unclear. This study explores the effects of Gal‐3 on ox‐LDL‐induced endothelial dysfunction and the underlying molecular mechanisms. In this study, Gal‐3, integrin β1, and GTP‐RhoA in the blood and plaques of AS patients were examined by ELISA and western blot respectively. Their levels were found to be obviously upregulated compared with non‐AS control group. CCK8 assay and flow cytometry analysis showed that Gal‐3 significantly decreased cell viability and promoted apoptosis in ox‐LDL‐treated human umbilical vascular endothelial cells (HUVECs). The upregulation of integrinβ1, GTP‐RhoA, p‐JNK, p‐p65, p‐IKKα, and p‐IKKβ induced by ox‐LDL was further enhanced by treatment with Gal‐3. Pretreatment with Gal‐3 increased expression of inﬂammatory factors (interleukin [IL]‐6, IL‐8, and IL‐1β), chemokines(CXCL‐1 and CCL‐2) and adhesion molecules (VCAM‐1 and ICAM‐1). Furthermore, the promotional effects of Gal‐3 on NF‐κB activation and inflammatory factors in ox‐LDL‐treated HUVECs were reversed by the treatments with integrinβ1‐siRNA or the JNK inhibitor. We also found that integrinβ1‐siRNA decreased the protein expression of GTP‐RhoA and p‐JNK, while RhoA inhibitor partially reduced the upregulated expression of p‐JNK induced by Gal‐3. In conclusion, our finding suggests that Gal‐3 exacerbates ox‐LDL‐mediated endothelial injury by inducing inflammation via integrin β1‐RhoA‐JNK signaling activation.

## 1 INTRODUCTION

Endothelial cells (ECs) play a key role in maintaining vascular homeostasis in response to various stimuli. Endothelial dysfunction in arterial walls has been considered to be the basis and initial step of atherosclerosis (AS), the most common cause in cardiovascular diseases (Davignon & Ganz, 2004; Tabas, García‐Cardeña, & Owens, 2015). Increasing evidence has demonstrated that oxidized low‐density lipoprotein (ox‐LDL) induces apoptosis of endothelial cells and is considered as the major risk factor in the progression of AS(J. Li, Liang, Wang, Xu, & Li, 2017; Qin et al., 2017; Veas et al., 2016). To date, the underlying molecular mechanisms of AS progression are complicated and not completely understood.

Many studies have demonstrated the role of inflammation in endothelial dysfunction(Cho, Lee, Chang, Lee, & Kim, 2018; Chrysohoou, Kollia, & Tousoulis, 2018; Stolberg et al., 2018). Under conditions of chronic inflammation, sustained activation of ECs by inflammatory stimuli causes alterations in normal endothelial function, resulting in endothelial dysfunction. Accumulating evidence shows that ox‐LDL induces endothelial cell injury by changing pro‐inflammatory genes expression (J. B. Li et al., 2017; S. Zhang et al., 2017). In addition, several studies have demonstrated that ox‐LDL stimulates vascular cell adhesion molecule‐1 (VCAM‐1) and intercellular cell adhesion molecule‐1 (ICAM‐1) to exacerbate ECs dysfunction(Wang, Hao, Wang, Wang, & Li, 2011; H. Zhang, Zheng, Zhao, Guo, & Chen, 2013). However, the effective regulators and accurate mechanisms of inflammation in ox‐LDL induced AS remain to be thoroughly investigated.

As a multifunctional protein, Galectin‐3 (Gal‐3) is broadly expressed in inflammatory cells to induce the functions of macrophage chemotaxis, angiogenesis, lipid loading, and inflammation responding to biochemical and biophysical stimuli. An elevated level of Gal‐3 has been found to be significantly associated with higher risk of death in both acutely decompensated heart failure and chronic heart failure populations (Lok et al., 2010; van Kimmenade et al., 2006). Some studies have indicated that Gal‐3 inhibition significantly reduces hypertension and fibrosis (Martínez‐Martínez et al., 2018; Yu et al., 2013). The role of Gal‐3 in promoting AS has recently received great interest. Gal‐3 participates in different mechanisms involved in AS, such as inflammation, proliferation, or macrophage chemotaxis. However, the role and related mechanisms of Gal‐3 in ox‐LDL induced AS have not been reported.

A large number of basic and clinical studies have shown that Gal‐3 is mainly involved in the formation and development of AS by exacerbating the inflammation of coronary arteries (Madrigal‐matute et al., 2014). Integrins are transmembrane receptors that facilitate cell‐extracellular matrix adhesion and signal transduction. Some studies have shown that Gal‐3 can interact with integrin β1 (Hönig et al., 2018; Nachtigal, Ghaffar, & Mayer, 2008; Kianoush, F., 2017) and indicated that integrin β1 activates RhoA, thus upregulating JNK signaling, an important branch of the MAPK (mitogen‐activated protein kinase), which plays an important role in many physiological and pathological processes,such as cell stress, inflammation and apoptosis (Wang et al., 2016; L. Zhang et al., 2017).

JNK, a common receptor tyrosine kinase, participates in wide ranges of physiological processes. Recent reports also indicate that JNK produces a molecular link between inflammation and AS (Kwok, K. H. M., 2016; Mammen et al., 2018). Hence, the activation of JNK can accelerate the formation of AS to some extent. In the present study, we hypothesize that the Gal‐3‐integrin β1 interaction may promote the expression of inflammatory factors and adhesion molecules by modulating the RhoA‐JNK signaling pathway, thus resulting in the progression of AS.

## 2 MATERIALS AND METHODS

### 2.1 Collection of human specimens

Blood samples were obtained from people diagnosed with color Doppler ultrasound in Nanjing BenQ Hospital, affiliated to Nanjing Medical University. Specimens were collected from patients who underwent carotid endarterectomy (CEA) in The First Affiliated Hospital of Nanjing Medical University. The splenic arteries in the control group were from traumatic splenectomy. The study was approved by the ethics committee of Nanjing BenQ Hospital and The First Affiliated Hospital of Nanjing Medical University. The protein levels in blood and CEA specimens were analyzed by enzyme‐linked immunosorbent assay (ELISA) and western blot (WB), respectively.

### 2.2 Materials

Ox‐LDL was purchased from Solarbio (Beijing, China). Gal‐3 was purchased from Peprotech (BioGems, Rocky Hill, NJ). The ELISA kits of interleukin (IL)‐6, IL‐8, IL‐1β, CXCL‐1, and CCL‐2 were purchased from MultiSciences (Lianke, Hangzhou, China). GTP‐RhoA specific inhibitor Y‐27632 and JNK inhibitor SP600125 were purchased from Targetmol (Target Molecule Corp, Shanghai, China)

### 2.3 Cell culture

HUVECs were purchased from Type Culture Collection of the Chinese Academy of Sciences (Shanghai, China). They were cultured in Dulbecco's modiﬁed Eagle's medium (DMEM, Thermo Fisher Scientiﬁc) supplemented with 10% fetal bovine serum, 100 U/ml penicillin Gand 100 mg/ml streptomycin at 37°C in a humidiﬁed atmosphere containing 5% CO_2_.

### 2.4 Cell groups division

The HUVECs were first divided into four groups: (a) control group; (b) Gal‐3 group, cells treated with 250 ng/ml Gal‐3 for 48 hr; (c) ox‐LDL group, cells treated with 150 μg/ml ox‐LDL for 6 hr; (d) Gal‐3 + ox‐LDL group, cells treated with 250 ng/ml Gal‐3 for 48 hr before treatment of ox‐LDL for 6 hr. Furthermore, cells were further divided into five groups: (a) ox‐LDL group; (b) Gal‐3 + ox‐LDL group; (c) control‐siRNA + Gal‐3 (250 ng/ml + ox‐LDL (150 μg/ml); (d) integrin β1‐siRNA + Gal‐3 (250 ng/ml) + ox‐LDL (150 μg/ml), cells transfected with integrin β1 for 72 hr before treatment of 250 ng/ml Gal‐3 for 48 hr, followed by treatment with 150 μg/ml ox‐LDL for 6 hr; (e) JNK inhibitor (SP600125) + Gal‐3 + ox‐LDL, cells treated with 20 μM SP600125 for 1 hr before treatment with 250 ng/ml Gal‐3 for 48 hr, followed by treatment with 150 μg/ml ox‐LDL for 6 hr.

### 2.5 Cell viability analysis

The viability of HUVECs was evaluated by 3‐(4,5‐dimethylthiazol‐2‐yl)‐2, 5‐diphenyltetrazolium bromide (MTT) assays. HUVECs were seeded in a 96‐well plate and treated according to the requirements of the different groups. The cells were treated with 100 μg/ml MTT for 4 hr in a 37°C incubator. Subsequently, dimethyl sulfoxide (DMSO, 200 ml/well) was added to dissolve the formazan crystals. The absorbance at 490 nm was detected using a microplate reader (Bio‐Rad, Inc, Hercules, CA).

### 2.6 Annexin V‐FITC/propidium iodide assay

HUVECs were seeded in a 6‐well plate and treated according to the requirements of the different groups. Apoptosis was determined by ﬂow cytometry. In summary, the differently treated cells were harvested and washed three times with cold phosphate‐buffered saline (PBS). Subsequently, 10^6^ cells were resuspended in the binding buffer (500 ml) and incubated with Annexin‐V‐FITC (10 ml) and propidium iodide (PI) (5 ml) for 15 min at 37°C in the dark. Finally, the ﬂuorescent intensities were detected with a FACSCalibur™ flow cytometer (Becton Dickinson, San Jose, CA, USA) and apoptosis rates were analyzed within 1 hr.

### 2.7 WB analysis

HUVECs were lysed in RIPA buffer. Equivalent amounts of total protein (20 mg) were separated by 10% SDS‐PAGE and then transferred to polyvinylidene ﬂuoride (PVDF) membrane. The membrane was blocked in TBST containing 5% nonfat milk for 2 hr at room temperature. Subsequently, the membranes were incubated with the following primary antibodies at 4°C overnight: integrin β1 (1:1000, cat.24693, abcam), RhoA (1:1000, cat.6352, Affinity), phosphorylation of RhoA (GTP‐RhoA) (1:500, cat.211164, abcam), JNK (1:1000, cat. AF6319, Affinity), p‐JNK (1:1000, cat. AF3320, Affinity), ICAM‐1 (1:1000, cat. DF7413, Affinity) and VCAM‐1 (1:1000, cat.DF6082, Affinity), NF‐κB P65(1:1000, AF5006, Affinity), PhosphoNFκB P65(1:1000, AF2006, Affinity), IKKα (1:1000, AF6012, Affinity), IKKβ (1:1000, bs‐4880R, Bioss, China), Phospho‐IKKβ(1:1000, bs‐3232R, Bioss, China). Glyceraldehyde 3‐phosphate dehydrogenase (GAPDH) was used as loading control. After incubation with horseradish peroxidase‐conjugated secondary antibodies, the blots were visualized using enhanced chemiluminescence kit (ECL, Perkin Elmer Life Sciences, Boston, MA). The bands were quantiﬁed and analyzed using the ImageJ software (National Institutes of Health, Bethesda, MD) and were normalized to GAPDH.

### 2.8 Cell staining for immunofluorescence microscopy

The treated cells were grown on coverslips, fixed with 3% paraformaldehyde for 15 min and permeabilized with 0.5% Triton X‐100 in PBS for 10 min at room temperature and blocked with 3% goat serum for 20 min at room temperature. Then cells were incubated with integrin β1 antibody (1:500, abcam) overnight at 4°C. The next day, cells were washed in 0.1 mol/L PBS for 3 times and then incubated with fluorescein‐conjugated affinipure goat antirabbit IgG (1:500) for 3 hr at 37°C and subsequently stained the nuclei with DAPI. Finally, cells were observed using an Olympus BX51 fluorescence microscope (Tokyo, Japan).

### 2.9 Small interfering RNA assay

5 × 10^5^ cells/ml cells were seeded in a 6‐well plate and incubated for 24 hr. Plasmid and small interfering RNA (siRNA) transfection were carried out using Lipofectamine 2000 (Invitrogen) following the manufacturer's instructions, when the cell confluence reached approximately 70%. Integrin‐β1‐siRNA (target sequence 5′‐CCACAGACAUUUACAUUAAUU‐ 3′) and negative control (NC) siRNA (HP GenomeWide siRNA; Qiagen, Hilden, Germany) were prepared and transfected at 10 nM for 48 hr. Subsequently, the transfected cells were analyzed by WB.

### 2.10 Enzyme‐linked immunosorbent assay

Supernatant of HUVECs of different groups were detected using corresponding enzyme‐linked immunosorbent assay (ELISA) kits according to the manufacturer's instructions. The expression of Gal‐3, using ELISA, was analyzed in eight blood specimens from patients with AS and eight blood specimens from the control group without AS. Furthermore, the expression of pro‐inﬂammatory cytokines (IL‐6, IL‐8 and IL‐1β) and chemokines (CXCL‐1 and CCL‐2) was assessed as well.

### 2.11 Statistical analysis

Data were presented as the mean ± SD. Differences between groups were analyzed by one‐way analysis of variance using GraphPad Prism 5.0 (GraphPad Software, San Diego, CA). *P* < 0.05 was considered to indicate statistical significance.

## 3 RESULTS

### 3.1 Protein expression of gal‐3, integrin β1, RhoA, and GTP‐RhoA in AS

**Figure 1:**
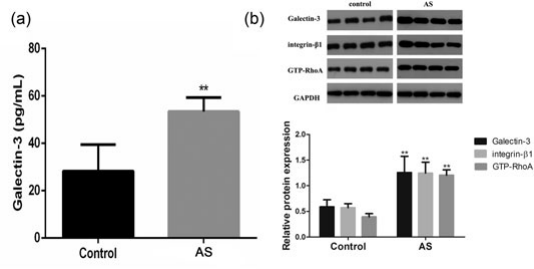
Protein expression of Gal‐3, integrin β1, RhoA, GTP‐RhoA in AS and some paired normal segments. (a) ELISA was used to detect the level of Gal‐3 in plasma. (b) Protein expression of Gal‐3, integrin β1 and GTP‐RhoA was assessed by WB and the relative protein expression was further indicated using histograms. **p < 0.01 versus control

We assessed the protein level of Gal‐3, using ELISA, and found Gal‐3 expression to be significantly higher in blood specimens from AS patients. (Figure 1a) Furthermore, the protein expression of Gal‐3, integrin β1, RhoA and GTP‐RhoA in the tissue of 4 AS plagues and four normal splenic artery was determined by WB. Gal‐3, integrin β1, and GTP‐RhoA levels were markedly elevated in AS tissue compared with the control group, (Figure 1b) while the RhoA level underwent almost no change. The results showed that although Gal‐3, integrin β1 and GTP‐RhoA were expressed in both normal and abnormal conditions, they were prominent in AS vessels. Considering this, we further investigated the role of Gal‐3 in the AS and the underlying molecular mechanisms among the three factors.

### 3.2 Galectin‐3 decreases viability and promotes cell apoptosis of ox‐LDL‐treated HUVECs

**Figure 2:**
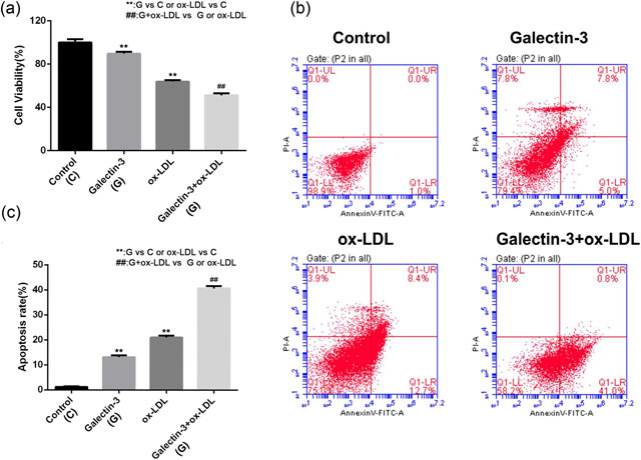
The effects of Gal‐3 on cell viability and apoptosis in ox‐LDL‐treated HUVECs. Cells were treated with 250 ng/ml Gal‐3 for 48 hr followed by treatment with ox‐LDL for 6 hr. (a) Cell viability was measured by the MTT assay. (b) Apoptosis was assessed by flow cytometry. (c) The rate of apoptosis was indicated using histograms. Each experiment was performed in triplicate. **p < 0.01, ^##^ *p* < 0.01 [Color figure can be viewed at wileyonlinelibrary.com]

To investigate the effects of Gal‐3 on the cell viability in ox‐LDL‐induced HUVECs, the cells were exposed to 250 ng/ml Gal‐3, 150 μg/ml ox‐LDL or their combination and the MTT assay was performed. We further measured apoptosis by ﬂow cytometry. As shown in Figure 2a, ox‐LDL treatment signiﬁcantly reduced cell viability as compared with the control group. The ox‐LDL‐induced reduction in cell viability was strengthened by Gal‐3. As shown in Figure 2b,c proportion of apoptotic cells was markedly increased in the ox‐LDL group, which was further significantly improved by additional Gal‐3 treatment.

### 3.3 Gal‐3 promotes ox‐LDL‐induced inflammatory responses via NF‐κB activation and enhances the expression of related inﬂammatory factors, chemokines, and adhesion molecules

**Figure 3:**
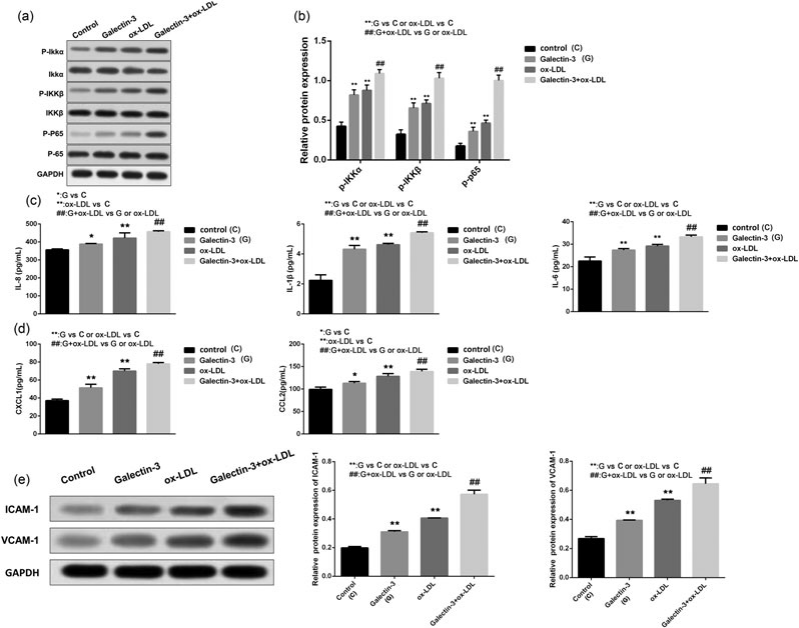
Gal‐3 promotes ox‐LDL‐induced inflammatory responses via NF‐κB activation and enhances the expression of related inﬂammatory factors, chemokines, and adhesion molecules. After exposure to Gal‐3, ox‐LDL or their combination, the total and phosphorylated expression of p65, IKKα, and IKKβ was examined by WB demonstrated by histogram (a and b); the levels of IL‐6, IL‐8, and IL‐1β and chemokines (CXCL‐1 and CCL‐2) were measured by ELISA. (c and d) The expression of VCAM‐1 and ICAM‐1 was detected by WB and the relative protein expression was given by histogram. (c) Each experiment was performed in triplicate. *p < 0.05, **p < 0.01, ^##^ *p* < 0.01

Activation of the NF‐κB pathway is widely recognized as characteristic of inflammation. To explore whether Gal‐3 aggravate ox‐LDL‐induced inflammatory responses in HUVECs, we first detected the expression of p65 NF‐κB after ox‐LDL or Gal‐3 alone or in combination, the result revealed that the single treatment obviously increased its expression and the effect of the combination was greater (Figure 3a,b). However, there was no change in the total protein of p65 NF‐κB. We further assessed whether NF‐κB activation induced by Gal‐3 was associated with the regulation effect of IKKα and IKKβ, two highly related IκB kinases. Similar to the above results, the phosphorylation levels of IKKα and IKKβ(p‐IKKα and p‐IKKβ) were significantly increased, and the combined effect was stronger(Figure 3a,b). Additionally, we also evaluated the effect of Gal‐3 on inflammatory factors. As shown in Figure 3c,d compared to the control group, the protein levels of pro‐inflammatory factors (IL‐6, IL‐8, and IL‐1β and chemokines (CXCL‐1 and CCL‐2), using ELISA, were markedly increased following ox‐LDL treatment. Moreover, the expression of these cytokines was signiﬁcantly increased in the combination of Gal‐3 and ox‐LDL group. As shown in Figure 3e, the adhesion molecules VCAM‐1 and ICAM‐1 were notably upregulated in the ox‐LDL group compared to the control group, and their expression was significantly elevated by the addition of Gal‐3 treatment. These results suggest that Gal‐3 promotes HUVECs injury via ox‐LDL‐induced inﬂammation.

### 3.4 Galectin‐3 promotes the expression of integrin β1, GTP‐RhoA and p‐JNK in ox‐LDL induced HUVECs

**Figure 4:**
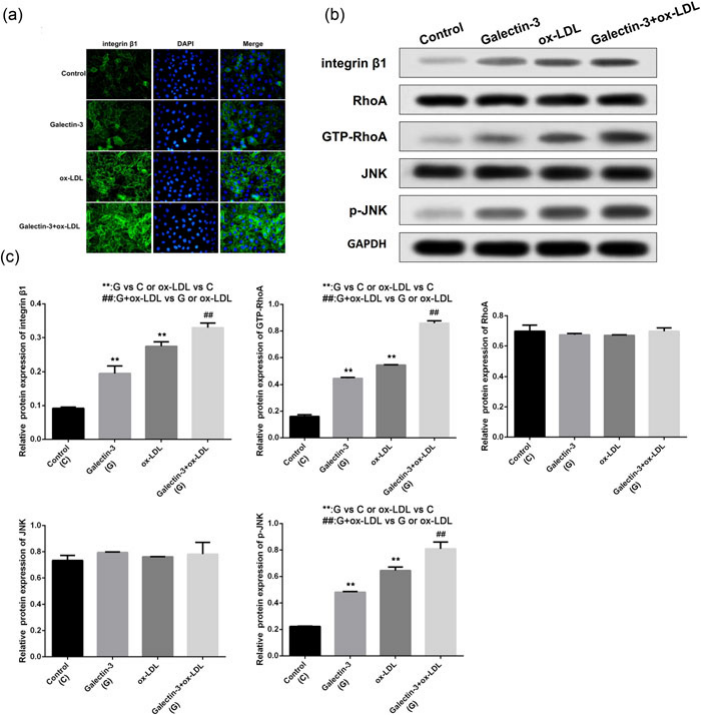
Gal‐3 increases the expression of integrin β1, GTP‐RhoA and p‐JNK in ox‐LDL induced HUVECs. After exposure to Gal‐3, ox‐LDL or their combination, the expression of integrin β1 was detected by IF, (a) WB was used to measure the protein levels of integrin β1, RhoA, JNK, GTP‐RhoA, and p‐JNK (b). The relative protein expression was further indicated using histograms (c). **p < 0.01, ^##^ *p* < 0.01 [Color figure can be viewed at wileyonlinelibrary.com]

To determine whether integrin β1 was involved in the effect of Gal‐3, integrin β1 protein was measured by IF. Integrin β1 was moderately increased following exposure to Gal‐3 or ox‐LDL alone. However, a combination of them resulted in significantly higher expression as compared to single treatment. (Figure 4a) We also detected the change of integrin β1 protein expression using WB and the result was consistent with IF. (Figure 4b) In addition, to test whether RhoA or JNK molecule was involved in Gal‐3 effect, the levels of RhoA, JNK, GTP‐RhoA and p‐JNK were further assessed after Gal‐3 and ox‐LDL treatment. The result indicated that single treatment obviously enhanced the levels of GTP‐RhoA and p‐JNK in the HUVECs cells, whereas their total levels remained unchanged. Moreover, treatment of Gal‐3 combined with ox‐LDL induced higher GTP‐RhoA and p‐JNK expression as compared to single drug treatment. (Figure 4c)

### 3.5 Galectin‐3 aggravates ox‐LDL induced HUVECs injury by activating integrin β1‐RhoA‐JNK pathway

**Figure 5:**
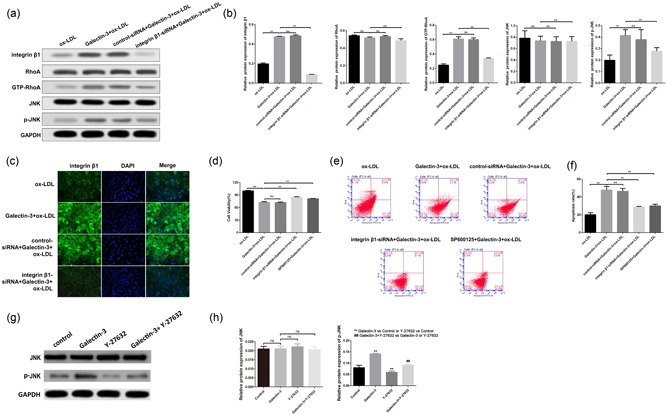
Gal‐3 aggravates ox‐LDL induced HUVECs injury by activating integrin β1‐RhoA‐JNK pathway. HUVECs, treated by ox‐LDL alone or in combination with Gal‐3, were performed to knockdown integrin β1 by siRNA, and the levels of integrin β1 and relative factors (JNK, pJNK, RhoA and GTP‐RhoA) were examined by WB and demonstrated by histogram (A and B). IF was performed to assess the expression of integrin β1 after knockdown. (c) Cell viability was measured by MTT assay (d) and apoptosis was determined by flow cytometry (E and F) using siRNA or JNK inhibitor SP600125. The changes of JNK and p‐JNK expression were evaluated after the treatment of RhoA inhibitor Y‐27632 alone or in combination with Gal‐3. (G and H) **p < 0.01, ^##^ *p* < 0.01 [Color figure can be viewed at wileyonlinelibrary.com]

To investigate the role of integrin β1‐RhoA‐JNK pathway in the ox‐LDL induced HUVECs cell injury, we knocked down integrin β1 using siRNA in ox‐LDL‐treated HUVECs and the decreased integrin β1 expression was confirmed by WB and IF (Figure 5a, b and c). Next, the MTT assay and ﬂow cytometry were performed to evaluate cell viability and apoptosis (Figure 5d, e and f). Integrin β1 knockdown and the JNK inhibitor, SP600125, signiﬁcantly increased cell viability and decreased the rate of cell apoptosis, suggesting reducing Gal‐3 induced HUVECs cell injury. Moreover, downregulation of integrin β1 markedly reduced GTP‐RhoA and p‐JNK. To explore whether RhoA was involved in the regulation of integrin β1 on JNK, we chose RhoA specific inhibitor, Y‐27632 to suppress its expression. We found that Y‐27632 markedly retarded JNK activation, while Gal‐3 promoted JNK expression. Meanwhile, Y‐27632 partially inhibited the upregulated expression of p‐JNK induced by Gal‐3. (Figure 5g,h) These results suggest that Gal‐3 promotes ox‐LDL induced HUVECs cell injury by activating integrin β1‐RhoA‐JNK pathway.

### 3.6 Gal‐3‐induced inflammatory pathway NF‐κB activation and expression of related cytokines, chemokines, and adhesion molecules play a promotional role in ox‐LDL‐induced HUVECs inflammatory injury by activating integrin β1‐RhoA‐JNK pathway

**Figure 6:**
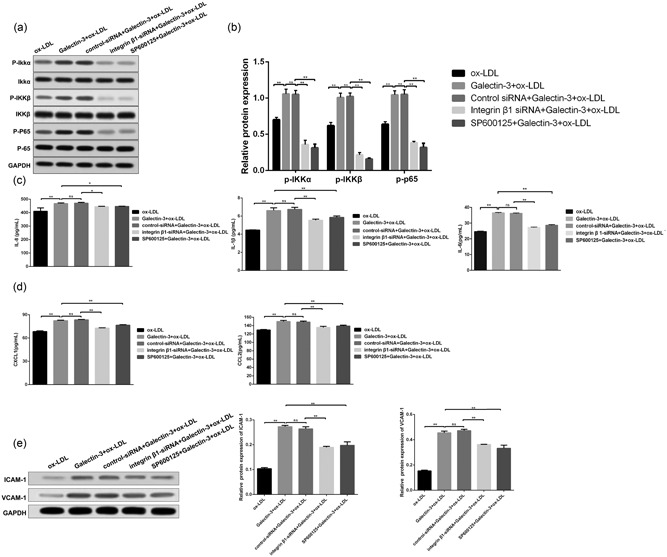
Gal‐3 induced expression of inflammatory factors promoted HUVECs injury via integrin β1‐RhoA‐JNK pathway. HUVECs, exposing to the ox‐LDL alone or in combination with Gal‐3 were further treated with integrin β1‐siRNA or JNK inhibitor (SP600125). The total and phosphorylated expression of p65,IKKα and IKKβ after the above treatment was examined by WB and demonstrated by histogram. (a and b).The levels of inflammatory cytokines and chemokines were then measured by ELISA. (c and d) The expression of adhesion molecules (ICAM‐1 and VCAM‐1) was detected by WB (e). *p < 0.05, **p < 0.01

To explore whether Gal‐3 induced HUVECs injury via **i**ntegrin β‐RhoA‐JNK pathway is associated with inﬂammation, we first assessed the inhibition of integrin β1 and JNK on NF‐κB activation, the results indicated that siRNA integrin β1 or SP600125 significantly inhibited the phosphorylation expression of p65, IKKα and IKKβ. (Figure 6a,b). Furthermore, the levels of pro‐inﬂammatory cytokines (IL‐6, IL‐8, and IL‐1β) and chemokines (CXCL‐1 and CCL‐2) were then assessed by ELISA. Data in Figure 6c,d demonstrated that integrin β1 knockdown or JNK inhibition signiﬁcantly decreased the level of IL‐6, IL‐8, IL‐1β, CXCL‐1, and CCL‐2. Moreover, either integrin β1 knockdown or JNK inhibition signiﬁcantly downregulated the expression of the adhesion molecules VCAM‐1 and ICAM‐1. (Figure 6e)

## 4 DISCUSSION

Endothelial cells play a key role in maintaining vascular homeostasis in response to various stimuli. However, vascular injuries, such as angioplasty, diabetes, hypertension, and immune‐mediated damages, can lead to endothelial cell dysfunction, which has been considered to be the ﬁrst cellular event in the pathogenesis of AS. Many studies have revealed an association between endothelial dysfunction and chronic inflammation (Cho et al., 2018; Chrysohoou et al., 2018; Stolberg et al., 2018). Endothelial dysfunction, because of inflammation, contributes to the pathogenesis of AS. Recent evidence suggests that Gal‐3 involves in the pathogenesis of inflammatory disorders (Knights et al., 2016; Lu et al., 2017; Oever et al., 2013) and the inactivation of Gal‐3 or therapeutic modulation of the protein levels has been shown to halt the progression of AS (Dumic, Dabelic, & Flögel, 2016; MacKinnon et al., 2013). In this study, we investigated the effects of Gal‐3 on endothelial injury. Our study is the first to show that Gal‐3 promotes ox‐LDL induced inflammation‐associated HUVECs injury by activating integrin β1‐RhoA‐JNK signaling pathway.

Gal‐3 has been shown to be involved in cell growth, differentiation, and apoptosis (Tian et al., 2015). Studies have also shown that the expression of Gal‐3 is implicated in a variety of processes associated with heart failure [13–14] and is responsible for the phenotypic transformation of HUVECs (Chen et al., 2016). However, little is currently known regarding the effects of Gal‐3 on ox‐LDL‐induced endothelial cell injury, which has been demonstrated to be closely related with AS.

In the present study, HUVECs were treated with ox‐LDL, Gal‐3 alone, or their combination. The results indicated that the additional Gal‐3 treatment markedly exacerbated HUVECs injury induced by ox‐LDL, according to the analysis of growth inhibition and apoptosis promotion. Furthermore, we found that knockdown of integrin β1, which has been proved to be the receptor of Gal‐3 (Wu et al., 2013), attenuated the above effect. Together, these results suggest that Gal‐3 aggravates effects on ox‐LDL‐induced endothelial injury by suppressing cell viability and promoting apoptosis.

It is now well established that inﬂammation, contributing to endothelial dysfunction, is the key causative underlying mechanistic player, at the molecular and cellular level, for the onset and development of subsequent inﬂammation‐related AS (Cho et al., 2018; Chrysohoou et al., 2018; J. B. Li et al., 2017; Stolberg et al., 2018; Wang et al., 2011; Zhang et al., 2013, 2017). Activation of the NF‐κB pathway is widely recognized as characteristic of inflammation. It is thought that NF‐κB activation is controlled by IKK complex, which can regulate its nuclear translocation and transactivation. IKKα and IKKβ mediate Ser536 phosphorylation in the p65 transactivation domain. Several studies have demonstrated that NF‐κB is involved in the development of various inflammatory cardiac pathologies, namely ischemic heart disease (Huang, Wen, Lin, Wei, & Huang, 2018), myocardial infarction (Maracle et al., 2018) and heart failure (Hu et al., 2018). In our study, our results indicated Gal‐3 effect was closely associated with the phosphorylation levels of p65, IKKα, and IKKβ levels, suggesting that Gal‐3 may be involved in HUVECs inflammatory reaction on IKK‐dependent NF‐κB activation. Furthermore, the expression of inﬂammatory cytokines, including inﬂammatory factors and adhesion molecules, can promote the adhesion and inﬁltration of monocytes to the vascular endothelium, leading to the activation of macrophages. The macrophages then absorb lipoprotein, resulting in foam cell formation, which further stimulates vascular inﬂammation. In the present study, the levels of pro‐inﬂammatory cytokines (IL‐6, IL‐8, and IL‐1β) and chemokines (CXCL‐1 and CCL‐2) were found to be signiﬁcantly increased by ox‐LDL treatment. This increase was further enhanced by Gal‐3 treatment. Our results also indicated that the expression of VCAM‐1 and ICAM‐1 in the ox‐LDL group was enhanced as compared with the control group, and the expression was further upregulated following Gal‐3 treatment. Additionally, the expression of VCAM‐1 and ICAM‐1 was found reduced after Gal‐3 activity inhibition. Together, these data suggest that Gal‐3 promotes ox‐LDL‐induced inflammatory responses.

JNK, which regulates activation of the inflammatory response, is critical in atherogenesis. Several studies have indicated Gal‐3 exhibits cytokine‐like regulatory action via the JNK pathway and plays an important role in tumor progression, mast cells function adjustment and antiviral pharmacology (Galle, Hansen‐Hagge, Wanner, & Seibold, 2016; N. Li, Chen, Zhao, & Wang, 2017; L. Zhang et al., 2017). To determine whether JNK was involved in the promotional effects of atherogenesis induced by Gal‐3, the WB results showed that ox‐LDL upregulated p‐JNK expression, which was further dramatically increased following Gal‐3 treatment. In addition, after blocking Gal‐3 activity, p‐JNK expression was significantly inhibited, demonstrating that JNK signaling may be involved in the development of AS induced by Gal‐3. We further investigated the effects of JNK signaling blocking on the endothelial injury. The results showed the crucial effects of Gal‐3, including the release of cytokines (IL‐6, IL‐8, and IL‐1β) and chemokines (CXCL‐1 and CCL‐2), were obviously reversed by p‐JNK inhibitor treatment. These results suggest that JNK plays an important role in Gal‐3‐induced endothelial injury partly by regulating inflammation.

We further explored how Gal‐3 affects JNK pathway mediated HUVECs injury. Gal‐3 has been reported to activate integrin β1 or JNK signaling pathway (Cardoso, Andrade, Bustos, & Chammas, 2016). Some studies have indicated that integrin β1 can activate RhoA activity, which further regulated the JNK signaling pathway (Verma et al., 2011). Some studies indicated that RhoA exerted an important influence on regulating ox‐LDL induced endothelial cell injury and AS (Galle, Hansen‐Hagge, Wanner, & Seibold, 2016; Li et al., 2017; Wang et al., 2016). In this study, the expression of integrin β1, GTP‐ RhoA, and p‐JNK was obviously increased after treatment with ox‐LDL, which was further enhanced by additional Gal‐3. Moreover, knockdown of integrin β1 dramatically decreased the GTP‐RhoA and p‐JNK levels. Our data further showed that the GTP‐RhoA inhibitor, Y‐27632, markedly downregulated the expression of p‐JNK and only partially reduced the effect of Gal‐3 on JNK expression, suggesting some other factors were involved in Gal‐3 induced HUVECs inflammatory injury besides RhoA, which will be further researched. Moreover, we also found the inhibition of integrin β1 or JNK obviously reduced cell injury, led to inhibition of NFkB inflammatory pathway and the decrease of related inflammatory factors in HUVECs. Together, our research suggests that Gal‐3 promotes ox‐LDL‐induced HUVECs injury by integrin β1‐RhoA‐JNK pathway.

In conclusion, this study is the first to demonstrate that Gal‐3 treatment significantly exacerbates ox‐LDL‐induced HUVECs injury. We also found that Gal‐3 promoted ox‐LDL‐induced inflammatory injury through activation of the integrin β1‐RhoA‐JNK pathway. However, this study has shortcomings. Our blood samples and CEA specimens were not abundant enough, and further in vivo experiments and more detection of human specimens may be better to explain this. Moreover, our future research is needed to determine the role of other factors besides inflammation in the pathogenic role of Gal‐3 in AS.

## CONFLICTS OF INTEREST

The authors declare that there are no conflicts of interest.

## References

[jcp27910-bib-0001] Cardoso, A. C. F. , Andrade, L. N. S. , Bustos, S. O. , & Chammas, R. (2016). Galectin‐3 determines tumor cell adaptive strategies in stressed tumor microenvironments. Frontiers in Oncology, 6, 127 10.3389/fonc.2016.00127 27242966 PMC4876484

[jcp27910-bib-0002] Chen, H. Y. , Sharma, B. B. , Yu, L. , Zuberi, R. , Weng, I. C. , Kawakami, Y. , … Liu, F. T. (2016). Role of galectin‐3 in mast cell functions: Galectin‐3‐deficient mast cells exhibit impaired mediator release and defective JNK expression. Journal of Immunology, 177, 4991–4997. 10.4049/jimmunol.177.8.4991 17015681

[jcp27910-bib-0003] Cho, J. G. , Lee, A. , Chang, W. , Lee, M. S. , & Kim, J. (2018). Endothelial to mesenchymal transition represents a key link in the interaction between inflammation and endothelial dysfunction. Frontiers in imunology, 20, 294 9. 10.3389/fimmu.2018.00294 PMC5826197 29515588

[jcp27910-bib-0004] Chrysohoou, C. , Kollia, N. , & Tousoulis, D. (2018). The link between depression and atherosclerosis through the pathways of inflammation and endothelium dysfunction. Maturitas, 109, 1–5. 10.1016/j.maturitas.2017.12.001 29452775

[jcp27910-bib-0005] Davignon, J. , & Ganz, P. (2004). Role of endothelial dysfunction in atherosclerosis. Circulation, 109, III27–III1132. 10.1161/01.CIR.0000131515.03336.f8 15198963

[jcp27910-bib-0006] Dumic, J. , Dabelic, S. , & Flögel, M. (2016). Galectin‐3: An open‐ended story. Biochimica et Biophysica Acta, 1760, 616–635. 10.1016/j.bbagen.2005.12.020 16478649

[jcp27910-bib-0007] Galle, J. , Hansen‐Hagge, T. , Wanner, C. , & Seibold, S. (2016). Impact of oxidized low density lipoprotein on vascular cells. Atherosclerosis, 185, 219–226. 10.1016/j.atherosclerosis.2005.10.005 16288760

[jcp27910-bib-0008] Hönig, E. , Ringer, K. , Dewes, J. , von Mach, T. , Kamm, N. , Kreitzer, G. , & Jacob, R. (2018). Galectin‐3 modulates the polarized surface delivery of β1‐integrin in epithelial cells. Journal of Cell Science, 2018, 131 10.1242/jcs.213199 29748377

[jcp27910-bib-0009] Hu, M. , Zhang, Z. , Liu, B. , Zhang, S. , Chai, R. , Chen, X. , … Liu, N. (2018). Deubiquitinase inhibitor auranofin attenuated cardiac hypertrophy by blocking NF‐κB activation. Cellular Physiology and Biochemistry, 45, 2421–2430. 10.1159/000488230 29554646

[jcp27910-bib-0010] Huang, W. Q. , Wen, J. L. , Lin, R. Q. , Wei, P. , & Huang, F. (2018). Effects of mTOR/NF‐κB signaling pathway and high thoracic epidural anesthesia on myocardial ischemia‐reperfusion injury via autophagy in rats. Journal of Cellular Physiology, 223, 6669–6678. 10.1002/jcp.26320 29206300

[jcp27910-bib-0011] Kianoush, F. , Nematollahi, M. , Waterfield, J. D. , & Brunette, D. M. (2017). Regulation of RAW264.7 macrophage polarization on smooth and rough surface topographies by galectin‐3. Journal of biomedical materials research. Part A, 105, 2499–2509. 10.1002/jbm.a.36107 28498622

[jcp27910-bib-0012] Knights, A. J. , Yik, J. J. , Mat Jusoh, H. , Norton, L. J. , Funnell, A. P. W. , Pearson, R. C. M. , … Quinlan, K. G. R. (2016). Krüppel‐like factor 3 (KLF3/BKLF) Is required for widespread repression of the inflammatory modulator galectin‐3. Journal of Biological Chemistry, 291, 16048–16058. 10.1074/jbc.M116.715748 27226561 PMC4965555

[jcp27910-bib-0013] Kwok, K. H. M. , Cheng, K. K. Y. , Hoo, R. L. C. , Ye, D. , Xu, A. , & Lam, K. S. L. (2016). Adipose‐specific inactivation of JNK alleviates atherosclerosis in apoE‐deficient mice. Clin Sci (Lond), 130, 2087–2100. 10.1042/CS20160465 27512097

[jcp27910-bib-0014] Li, J. , Liang, X. , Wang, Y. , Xu, Z. , & Li, G. (2017). Investigation of highly expressed PCSK9 in atherosclerotic plaques and ox‐LDL‐induced endothelial cell apoptosis. Molecular Medicine Reports, 16, 1817–1825. 10.3892/mmr.2017.6803 28656218 PMC5561783

[jcp27910-bib-0015] Li, J. B. , Wang, H. Y. , Yao, Y. , Sun, Q. F. , Liu, Z. H. , Liu, S. Q. , … Liu, H. Y. (2017). Overexpression of microRNA‐138 alleviates human coronary artery endothelial cell injury and inflammatory response by inhibiting the PI3K/Akt/eNOS pathway. Journal of Cellular and Molecular Medicine, 21, 1482–1491. 10.1111/jcmm.13074 28371277 PMC5542903

[jcp27910-bib-0016] Li, N. , Chen, J. , Zhao, J. , & Wang, T. (2017). MicroRNA‐3188 targets ETS‐domain protein and participates in RhoA/ROCK pathway to regulate the development of atherosclerosis. Pharmazie, 72, 687–693. 10.1691/ph.2017.7686 29442044

[jcp27910-bib-0017] Lok, D. J. A. , Van Der Meer, P. , de la Porte, P. W. B. A. , Lipsic, E. , Van Wijngaarden, J. , Hillege, H. L. , & van Veldhuisen, D. J. (2010). Prognostic value of galectin‐3, a novel marker of fibrosis, in patients with chronic heart failure: Data from the DEAL‐HF study. Clinical Research in Cardiology, 99, 323–328. 10.1007/s00392-010-0125-y 20130888 PMC2858799

[jcp27910-bib-0018] Lu, Y. , Zhang, M. , Zhao, P. , Jia, M. , Liu, B. , Jia, Q. , … Li, J. (2017). Modified citrus pectin inhibits galectin‐3 function to reduce atherosclerotic lesions in apoE‐deficient mice. Molecular Medicine Reports, 16, 647–653. 10.3892/mmr.2017.6646 28560429 PMC5482107

[jcp27910-bib-0019] MacKinnon, A. C. , Liu, X. , Hadoke, P. W. , Miller, M. R. , Newby, D. E. , & Sethi, T. (2013). Inhibition of galectin‐3 reduces atherosclerosis in apolipoprotein E‐deficient mice. Glycobiology, 23, 654–663. 10.1093/glycob/cwt006 23426722 PMC3641797

[jcp27910-bib-0020] Madrigal‐matute, J. , Lindholt, J. S. , Fernandez‐garcia, C. E. , Benito‐martin, A. , Burillo, E. , Zalba, G. , … Martin‐Ventura, J. L. (2014). Galectin‐3, a biomarker linking oxidative stress and inflammation with the clinical outcomes of patients with atherothrombosis. Journal of the American Heart Association, 3, e000785. 10.1161/JAHA.114.000785 PMC4310363 25095870

[jcp27910-bib-0021] Mammen, M. J. , Sands, M. F. , Abou‐Jaoude, E. , Aalinkeel, R. , Reynolds, J. L. , Parikh, N. U. , … Mahajan, S. D. (2018). Role of Galectin‐3 in the pathophysiology underlying allergic lung inflammation in a tissue inhibitor of metalloproteinases 1 knockout model of murine asthma. Immunology, 153, 387–396. 10.1111/imm.12848 28992358 PMC5795177

[jcp27910-bib-0022] Maracle, C. X. , Agca, R. , Helder, B. , Meeuwsen, J. A. L. , Niessen, H. W. M. , Biessen, E. A. L. , … Tas, S. W. (2018). Noncanonical NF‐κB signaling in microvessels of atherosclerotic lesions is associated with inflammation, atheromatous plaque morphology and myocardial infarction. Atherosclerosis, 270, 33–41. 10.1016/j.atherosclerosis.2018.01.032 29407886

[jcp27910-bib-0023] Martínez‐Martínez, E. , Ibarrola, J. , Fernández‐Celis, A. , Calvier, L. , Leroy, C. , Cachofeiro, V. , … López‐Andrés, N. (2018). Galectin‐3 pharmacological inhibition attenuates early renal damage in spontaneously hypertensive rats. Journal of Hypertension, 36, 368–376. 10.1097/HJH.0000000000001545 28858976

[jcp27910-bib-0024] Nachtigal, M. , Ghaffar, A. , & Mayer, E. P. (2008). Galectin‐3 gene inactivation reduces atherosclerotic lesions and adventitial inflammation in ApoE‐deficient mice. American Journal of Pathology, 172, 247–255. 10.2353/ajpath.2008.070348 18156214 PMC2189631

[jcp27910-bib-0025] Oever, J. , Giamarellos‐Bourboulis, E. J. , Veerdonk, F. L. , Stelma, F. F. , Simon, A. , Janssen, M. , … Netea, M. G. (2013). Circulating galectin‐3 in infections and non‐infectious inflammatory diseases. European Journal of Clinical Microbiology and Infectious Diseases, 32, 1605–1610. 10.1007/s10096-013-1919-4 23828453

[jcp27910-bib-0026] Qin, B. , Shu, Y. , Xiao, L. , Lu, T. , Lin, Y. , Yang, H. , & Lu, Z. (2017). MicroRNA‐150 targets ELK1 and modulates the apoptosis induced by ox‐LDL in endothelial cells. Molecular and Cellular Biochemistry, 429, 45–58. 10.1007/s11010-016-2935-3 28110404

[jcp27910-bib-0027] Stolberg, C. R. , Mundbjerg, L. H. , Funch‐Jensen, P. , Gram, B. , Bladbjerg, E. M. , & Juhl, C. B. (2018). Effects of gastric bypass surgery followed by supervised physical training on inflammation and endothelial function: A randomized controlled trial. Atherosclerosis, 273, 37–44. 10.1016/j.atherosclerosis.2018.04.002 29677629

[jcp27910-bib-0028] Tabas, I. , García‐Cardeña, G. , & Owens, G. K. (2015). Recent insights into the cellular biology of atherosclerosis. Journal of Cell Biology, 209, 13–22. 10.1083/jcb.201412052 25869663 PMC4395483

[jcp27910-bib-0029] Tian, L. , Chen, K. , Cao, J. , Han, Z. , Gao, L. , Wang, Y. , … Wang, C. (2015). Galectin‐3‐induced oxidized low‐density lipoprotein promotes the phenotypic transformation of vascular smooth muscle cells. Molecular Medicine Reports, 12, 4995–5002. 10.3892/mmr.2015.4075. 26165519 PMC4581830

[jcp27910-bib-0030] van Kimmenade, R. R. , Januzzi, J. L. , Ellinor, P. T. , Sharma, U. C. , Bakker, J. A. , Low, A. F. , … Pinto, Y. M. (2006). Utility of amino‐terminal pro‐brain natriuretic peptide, galectin‐3, and apelin for the evaluation of patients with acute heart failure. Journal of the American College of Cardiology, 48, 1217–1224. 10.1016/j.jacc.2006.03.061 16979009

[jcp27910-bib-0031] Veas, C. , Jara, C. , Willis, N. D. , Pérez‐Contreras, K. , Gutierrez, N. , Toledo, J. , … Aguayo, C. (2016). Overexpression of LOXIN protects endothelial progenitor cells from apoptosis induced by oxidized low density lipoprotein. Journal of Cardiovascular Pharmacology, 67, 326–335. 10.1097/FJC.0000000000000358 26771151

[jcp27910-bib-0032] Verma, S. K. , Lal, H. , Golden, H. B. , Gerilechaogetu, F. , Smith, M. , Guleria, S. , … Dostal, D. E. (2011). Rac1 and RhoA differentially regulate angiotensinogen gene expression in stretched cardiac fibroblasts. Cardiovascular Research, 90, 88–96. 10.1093/cvr/cvq385 21131638 PMC3058736

[jcp27910-bib-0033] Wang, L. , Hao, Q. , Wang, Y. D. , Wang, W. J. , & Li, D. J. (2011). Protective effects of dehydroepiandrosterone on atherosclerosis in ovariectomized rabbits via alleviating inflammatory injury in endothelial cells. Atherosclerosis, 214, 47–57. 10.1016/j.atherosclerosis.2010.07.043 21071029

[jcp27910-bib-0034] Wang, L. , Luo, J. Y. , Li, B. , Tian, X. Y. , Chen, L. J. , Huang, Y. , … Huang, Y. (2016). Integrin‐YAP/TAZ‐JNK cascade mediates atheroprotective effect of unidirectional shear flow. Nature, 540, 579–582. 10.1038/nature20602 27926730

[jcp27910-bib-0035] Wu, K. L. , Huang, E. Y. , Jhu, E. W. , Huang, Y. H. , Su, W. H. , Chuang, P. C. , & Yang, K. D. (2013). Overexpression of galectin‐3 enhances migration of colon cancer cells related to activation of the K‐Ras‐Raf‐Erk1/2 pathway. Journal of Gastroenterology, 48, 350–359. 10.1007/s00535-012-0663-3 23015305

[jcp27910-bib-0036] Yu, L. , Ruifrok, W. P. T. , Meissner, M. , Bos, E. M. , van Goor, H. , Sanjabi, B. , … de Boer, R. A. (2013). Genetic and pharmacological inhibition of galectin‐3 prevents cardiac remodeling by interfering with myocardial fibrogenesis. Circulation: Heart Failure, 6, 107–117. 10.1161/CIRCHEARTFAILURE.112.971168 23230309

[jcp27910-bib-0037] Zhang, H. , Zheng, F. , Zhao, J. , Guo, D. , & Chen, X. (2013). Genistein inhibits ox‐LDL‐induced VCAM‐1, ICAM‐1 and MCP‐1 expression of HUVECs through heme oxygenase‐1. Archives of Medical Research, 44(2013), 13–20. 10.1016/j.arcmed.2012.12.001 23291378

[jcp27910-bib-0038] Zhang, L. , Wang, P. , Qin, Y. , Cong, Q. , Shao, C. , Du, Z. , … Ding, K. (2017). RN1, a novel galectin‐3 inhibitor, inhibits pancreatic cancer cell growth in vitro and in vivo via blocking galectin‐3 associated signaling pathways. Oncogene, 36(2017), 1297–1308. 10.1038/onc.2016.306 27617577

[jcp27910-bib-0039] Zhang, S. , Guo, C. , Chen, Z. , Zhang, P. , Li, J. , & Li, Y. (2017). Vitexin alleviates ox‐LDL‐mediated endothelial injury by inducing autophagy via AMPK signaling activation. Molecular Immunology, 85, 214–221. 10.1016/j.molimm.2017.02.020 28288411

